# Selection for Mitochondrial Quality Drives Evolution of the Germline

**DOI:** 10.1371/journal.pbio.2000410

**Published:** 2016-12-20

**Authors:** Arunas L. Radzvilavicius, Zena Hadjivasiliou, Andrew Pomiankowski, Nick Lane

**Affiliations:** 1 Centre for Mathematics and Physics in the Life Sciences and Experimental Biology, University College London, London, United Kingdom; 2 Research Department of Genetics, Evolution and Environment, University College London, London, United Kingdom; Newcastle University, United Kingdom

## Abstract

The origin of the germline–soma distinction is a fundamental unsolved question. Plants and basal metazoans do not have a germline but generate gametes from pluripotent stem cells in somatic tissues (somatic gametogenesis). In contrast, most bilaterians sequester a dedicated germline early in development. We develop an evolutionary model which shows that selection for mitochondrial quality drives germline evolution. In organisms with low mitochondrial replication error rates, segregation of mutations over multiple cell divisions generates variation, allowing selection to optimize gamete quality through somatic gametogenesis. Higher mutation rates promote early germline sequestration. We also consider how oogamy (a large female gamete packed with mitochondria) alters selection on the germline. Oogamy is beneficial as it reduces mitochondrial segregation in early development, improving adult fitness by restricting variation between tissues. But it also limits variation between early-sequestered oocytes, undermining gamete quality. Oocyte variation is restored through proliferation of germline cells, producing more germ cells than strictly needed, explaining the random culling (atresia) of precursor cells in bilaterians. Unlike other models of germline evolution, selection for mitochondrial quality can explain the stability of somatic gametogenesis in plants and basal metazoans, the evolution of oogamy in all plants and animals with tissue differentiation, and the mutational forces driving early germline sequestration in active bilaterians. The origins of predation in motile bilaterians in the Cambrian explosion is likely to have increased rates of tissue turnover and mitochondrial replication errors, in turn driving germline evolution and the emergence of complex developmental processes.

## Author Summary

Mammalian germ cells (eggs and sperm) are immortal in the sense that they propagate successive generations. In contrast, somatic (body) cells do not persist to the next generation. Yet neither plants nor basal animals such as sponges and corals have a germline; they simply form gametes from stem cells in adult tissues. The reasons for these differences are unknown. We develop an evolutionary model showing that the germline evolved in response to selection on mitochondria, the powerhouses of cells. Mitochondria retain their own genes, which occur in multiple copies per cell. In plants and basal animals, the mitochondrial genes mutate slowly. Segregation over the many rounds of cell division to form an adult generates variation in mutant mitochondria between gametes, sufficient for natural selection to improve mitochondrial function. In more active animals from the Cambrian explosion onwards, the mitochondrial mutation rate rose strongly. This required the evolution of a dedicated germline, set aside early in development, with lowered mutational input. It also favoured large eggs (starting with thousands of mitochondria) and culling, following overproduction (atresia). Both devices maintain mitochondrial quality. The evolution of germline sequestration had profound consequences, allowing the emergence of complex developmental processes and truly disposable adult tissues.

## Introduction

In distinguishing between the germline and soma, Weismann argued that the division of labour enabled the specialization of cells in somatic tissues, ultimately permitting greater organismal complexity [1]. In contrast, germline cells alone retain the capacity to provide genetic information for future generations and never form somatic cells [2]. Without the specialization enabled by the germline, complex multicellular animals with post-mitotic tissues such as brain might be impossible. But the division of labour cannot account for the origin of the germline, as all plants and many animals (including tunicates, flatworms, and Cnidaria [3]) have differentiated tissues but do not sequester a germline, instead generating gametes from pluripotent stem cells in somatic tissues (somatic gametogenesis).

The best-known hypothesis for the origin of germline sequestration relates to selfish competition between the cells of an individual. The strict distinction between germline and soma stabilises multicellular cooperation, as the only way for somatic cells to increase fitness is by cooperating with their kin in the germline [4–6]. According to this theory, plants did not evolve a germline because their rigid cell walls restrict cell movement, limiting the systemic effects of any parasitic cell lines [4]. In contrast, animal cells lack a rigid wall, making them more vulnerable to parasitic cell lines that undermine organismal function [4]. Sequestering a germline theoretically limits this competition. However, because cells in multicellular organisms normally derive from a single cell (unitary development), new selfish mutations must arise within a single generation; if these mutants are inherited then all cells in the offspring will carry the selfish mutation, so there are no longer any non-selfish cells to exploit [7,8]. The range of conditions under which selfish competition could give rise to the germline is therefore seriously restricted, if feasible at all [9].

A second line of thinking dating back to Weismann emphasises the protected environment of a sequestered germline [1,10]. By restricting mutations in gametes, the germline enhances germ-cell quality, contrasting with the “disposability” of the soma [11]. Avoiding the accumulation of mutations in nuclear genes associated with greater metabolic work can theoretically favour differentiation of a germline [12]. But testes have a high metabolic rate, and sperm are produced continuously through life (with 30 cell divisions by puberty and 400 divisions at the age of 30 in humans), contributing a large number of new nuclear mutations to offspring [13]. From this point of view, the male germline is equivalent to tissues such as bone marrow, merely specialising in the mass production of cells of a particular type. As all tissue precursor cells are spatially secluded—that is, sequestered—during early development in bilaterians, the male germline is distinct from other tissues only in that it produces gametes.

Focusing on the origin of the germline as a protected environment shifts the problem specifically to female gametogenesis. Across bilaterian animals, from nematode worms to mammals, primordial oocytes are sequestered early in development in a transcriptionally repressed state, with meiosis arrested in prophase I [14]. The mitochondria of primordial oocytes are held in a state of functional quiescence [15] before being amplified up to very large numbers during oocyte maturation, with as many as 10^6^ copies of mitochondrial DNA in mature mammalian oocytes [16]. Mitochondrial DNA is usually inherited uniparentally; male mitochondria are excluded from the oocyte or destroyed on entry to the zygote [17]. Taken together, these traits suggest that mitochondrial function might have played a role in germline evolution.

The possibility that selection for mitochondrial quality could have driven the evolution of two sexes, in which female mitochondria are protected by germline sequestration, has been raised before [15,18–20] but never formally modelled. Female germline mitochondria are proposed to act as “inert templates,” held in a metabolically quiescent state throughout development, whereas male germline mitochondria are damaged through use and so must be destroyed [15,18–20]. There are some difficulties with this hypothesis. First, mitochondria are typically inherited uniparentally (from one “sex”) even in isogamous organisms, where both gametes are motile, and so equally damaged through use [21,22]. Second, two sexes also exist in plants and basal metazoans, which produce oocytes and spermatozoa from the same stem cell lineage that gives rise to adult somatic cells, but do not sequester a germline [23]; no inert template mitochondria are sequestered early in development. Third, in bilaterians that do sequester a germline, most mitochondrial mutations seem to be produced by copying errors rather than reactive oxygen species (ROS) formed by respiration [24–27], so active use is only part of the problem. Finally, while female germline mitochondria seem to be inactive in some systems [15], they appear to be active in others, such as bivalve molluscs [28,29], and may generally be active in mature oocytes and during early development in mammals [30,31]. Overall, germline sequestration is plainly a secondary adaptation—uniparental inheritance and oogamy arose before oocyte sequestration in early development, and the evolution of two sexes cannot simply be a matter of protecting template mitochondria.

Here we investigate how early or later sequestration of gametes could improve mitochondrial quality. We propose that the key to germline evolution lies in the balance between mitochondrial mutations, which undermine fitness, and segregation of mutants, which generates variance, facilitating selection. Both are amplified with each round of cell division with the outcome depending on the relative influence of multiple factors. Our analysis reveals that selection for mitochondrial quality can explain many long-puzzling features of germline evolution, including the widespread stability of somatic gametogenesis in plants and basal metazoans, the secondary association between germline sequestration and complexity, the conditions required for early germline sequestration, and other peculiarities of female germline development, including extreme oogamy, follicular atresia, and the putative germline bottleneck.

## Results

### Mitochondrial Mutations, Segregation, and Fitness

**Fig 1:**
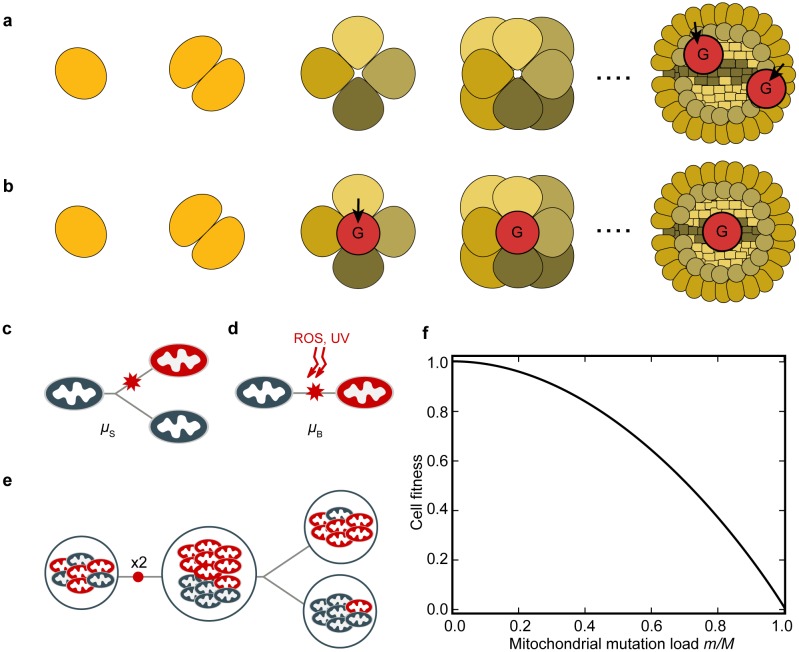
Life cycle of the model multicellular organism. **(a)** Development from zygote (left-hand side) to adult, showing early tissue differentiation (cells with differing shades) and late formation of gametes (G) from somatic cells in the adult (somatic gametogenesis). **(b)** Equivalent multicellular development depicting sequestration of gametes early in development (early germline). Dotted line indicates further development; adult and gamete cells not drawn to scale. **(c)** Copying errors (*μ*_S_) during replication of mitochondrial DNA. **(d)** Mutations caused by background damage (*μ*_B_) from, e.g., ultraviolet (UV) radiation or reactive oxygen species (ROS). **(e)** Doubling followed by random segregation of mitochondrial mutants (red) at cell division increases variance between daughter cells. **(f)** Concave fitness function, in which cell fitness declines non-linearly with the accumulation of mitochondrial mutations (*μ*_S_ + *μ*_B_) as seen in mitochondrial diseases (see text).

We consider an ancestral multicellular organism with isogamy that has evolved uniparental transmission of mitochondria exclusively from the “female” gamete (see Methods for full model details). Gametes are formed by meiosis late in development from the same stem cells that give rise to adult somatic cells, which we call somatic gametogenesis (Fig 1a). We then consider the selective conditions that favour the evolution of an early sequestered germline that has a reduced number of cellular replication cycles before gametes are produced (Fig 1b).

The quality of gametes and fitness of the organism depends on the number of mitochondrial mutations. These build up due to copying errors in mitochondrial genes at a rate *μ*_S_ per cell division (Fig 1c). Early sequestration of a germline restricts the number of cell divisions to gamete production and, therefore, the number of copying errors (Fig 1b). Mutations can also result from “background” damage, *μ*_B_, per unit time (caused by oxidative damage or UV radiation; Fig 1d), as cells can accumulate mitochondrial mutations even when not actively dividing [32–35]. So, background mutations affect gametes whether they are derived from an early germline or later from stem cells in adult tissues.

**Fig 2:**
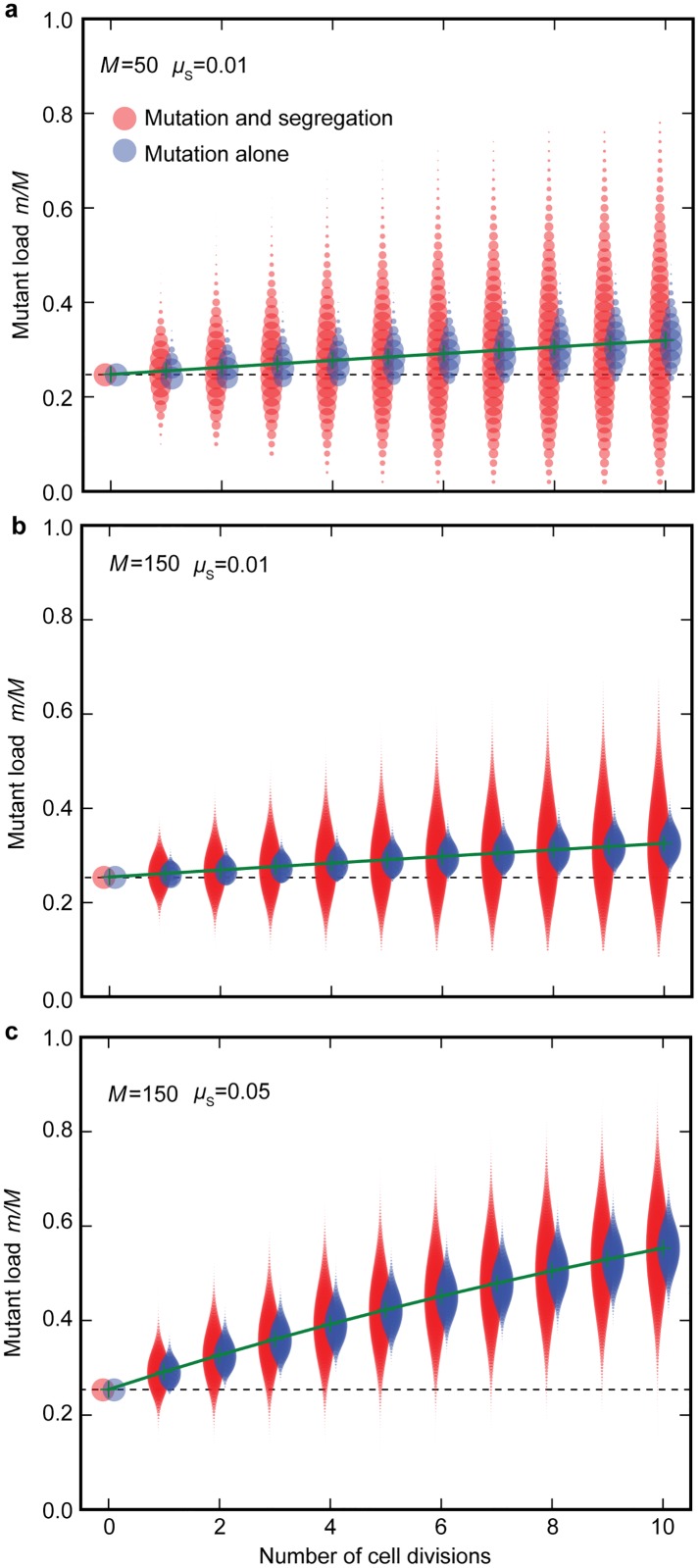
Segregational variance of mitochondrial mutants within a generation. At each cell division, the variance in mitochondrial mutant load (*m*/*M*) between daughter cells (red plots) increases due to mutational input as well as random segregation, generating cells with both more (above dotted line) and fewer (below dotted line) mutations than the zygotic cell. The blue plots show the variance in mutant load at each division due to mutation alone, without any random segregation. **(a)** The mutant load increases slowly with each cell division (green line) when the rate of copying errors is low (*μ*_S_ = 0.01). **(b)** Increasing mitochondrial number (*M*) decreases the variance in mutant load per cell (red plots) as the effects of random segregational drift are dampened. **(c)** Increasing copying errors (*μ*_S_ = 0.05) increases the mutant load in daughter cells; segregation no longer generates many high-quality daughter cells with fewer mutations than the zygote. Data were derived from a starting number of mutants in zygotes *m* = 0.24*M*, and then run iteratively through successive cell divisions as described in the first section of the Methods. Underlying data can be found at: https://github.com/ArunasRadzvilavicius/GermlineEvolution/tree/master/FigureData.

Unlike nuclear genes, which are clonally transmitted through mitosis, the mitochondrial population doubles and segregates at random to daughter cells at each cell division. Some daughter cells receive more, others fewer, mitochondrial mutations (Fig 1e). With somatic gametogenesis (i.e., late differentiation of germ cells), segregational variation has the capacity to generate gametes that carry few mutants or are even mutation free. The more rounds of cell division, the greater the degree of segregation, the higher the variance in mutation load, and the larger the proportion of gametes carrying very few or no mitochondrial mutations (Fig 2a). The effect of segregation is dampened with larger numbers of mitochondria per cell, as this diminishes drift (Fig 2b). At higher copying-error rates (high *μ*_S_), segregation still generates increased variance between daughter cells, but the proportion of gametes with few or no mutations falls to nearly zero (Fig 2c). For simplicity, we ignore selection at the level of mitochondria within cells, as we found that this roughly equates to changes in the mutation rate (as seen previously in [36,37]). For example, removing deleterious mitochondria by mitophagy effectively reduces the mutation rate, whereas faster replication of selfish or impaired mitochondria increases the mutation rate. Likewise, although the severity of mitochondrial mutations is variable, we fix each to have a similar effect for ease of analysis.

Overall, the resulting mitochondrial population in somatic cells is subject to a mutational load, which impacts on cell fitness

$$\omega \left(m\right)=1-s{\left(m/M\right)}^{2},$$ (1)

where *m* is the number of mutant mitochondria and *M* is the number of mitochondria in each cell. This fitness function is concave and assumes that a large number of mutants must accumulate before cell function is significantly undermined (Fig 1f). This is the case for mitochondrial diseases, in which mutant load can reach 60%–80% before symptoms become detectable—the well-known threshold effect [38,39]. We concentrate on a fitness function with *s* = 1, which is the case for large deletions, a common form of mitochondrial mutation [38,39]. Note that even when *s* = 1, a large number of mutations need to accumulate before there is a significant effect on cell fitness, and all mitochondria in the cell must mutate for cell fitness falls to zero. Other fitness functions (e.g., linear or convex, S1 Fig) and *s* < 1 (i.e., weaker selection, S2 Fig) do not affect the general outcome of our modelling; we do not pursue these alternative fitness functions further here, as they do not correspond well with known effects of mitochondrial mutations [38,39].

Another important dimension of fitness in multicellular organisms is the number and quality of tissues and their mutual interdependence. We take the fitness of a somatic tissue to be the mean fitness of its constituent cells, each of which reflects the accumulation and segregation of mutations as described above. We assume epistasis between tissues, so that adult fitness is determined by the quality of the worst tissue, while the magnitude of interdependence is in effect determined by the number of tissues (see Methods). If by chance a disproportionate number of mitochondrial mutations segregate into precursors of one tissue, the fitness of the adult is severely impaired; conversely, if mutations are distributed equally into the precursor cells of all tissues, organismal fitness is improved, as each tissue has equivalent quality. We also analysed an independent tissue epistasis parameter, which can be varied from zero to strong epistasis. The results are equivalent to those generated by varying tissue number, so we do not present them here.

Adult fitness thus depends on the number of mitochondrial mutations inherited by the zygote, the number of new mutations arising during development, how these mutations segregate into cells and tissues, and the degree of tissue-level epistasis. As we will show, this combination determines whether it is better to sequester germ cells early in development in a germline or derive them from adult somatic stem cells late in development. In the model, the specific mutation rates, number of rounds of cell division, mitochondrial numbers, and lifespan were chosen to illustrate the general forces operating and to be computationally tractable (e.g., a reasonably short lifespan, small numbers of cell divisions to adult life or germline sequestration, and relatively high mutation rates per cell division). Parameter values were not chosen to reflect particular species, which vary over many orders of magnitude. Importantly, we find that relative and not absolute values of model parameters matter, and we discuss our findings in this light.

### Germline Evolution Depends on Mitochondrial Mutation Rate, Not Complexity

The main benefit of early sequestration of germ cells is to reduce the number of cell divisions before gamete production, so reducing the net input of copying errors (*μ*_S_) in gametes. This raises the mean fitness of offspring in the next generation. But an early germline comes at the cost of reduced segregational variance. When gametes are derived later in development, there is more chance for segregation to generate gametes with lower numbers of mitochondrial mutations, which facilitates selection in the offspring, improving fitness over generations (Fig 2). The tension between these two forces determines the advantage (or disadvantage) of a germline.

**Fig 3:**
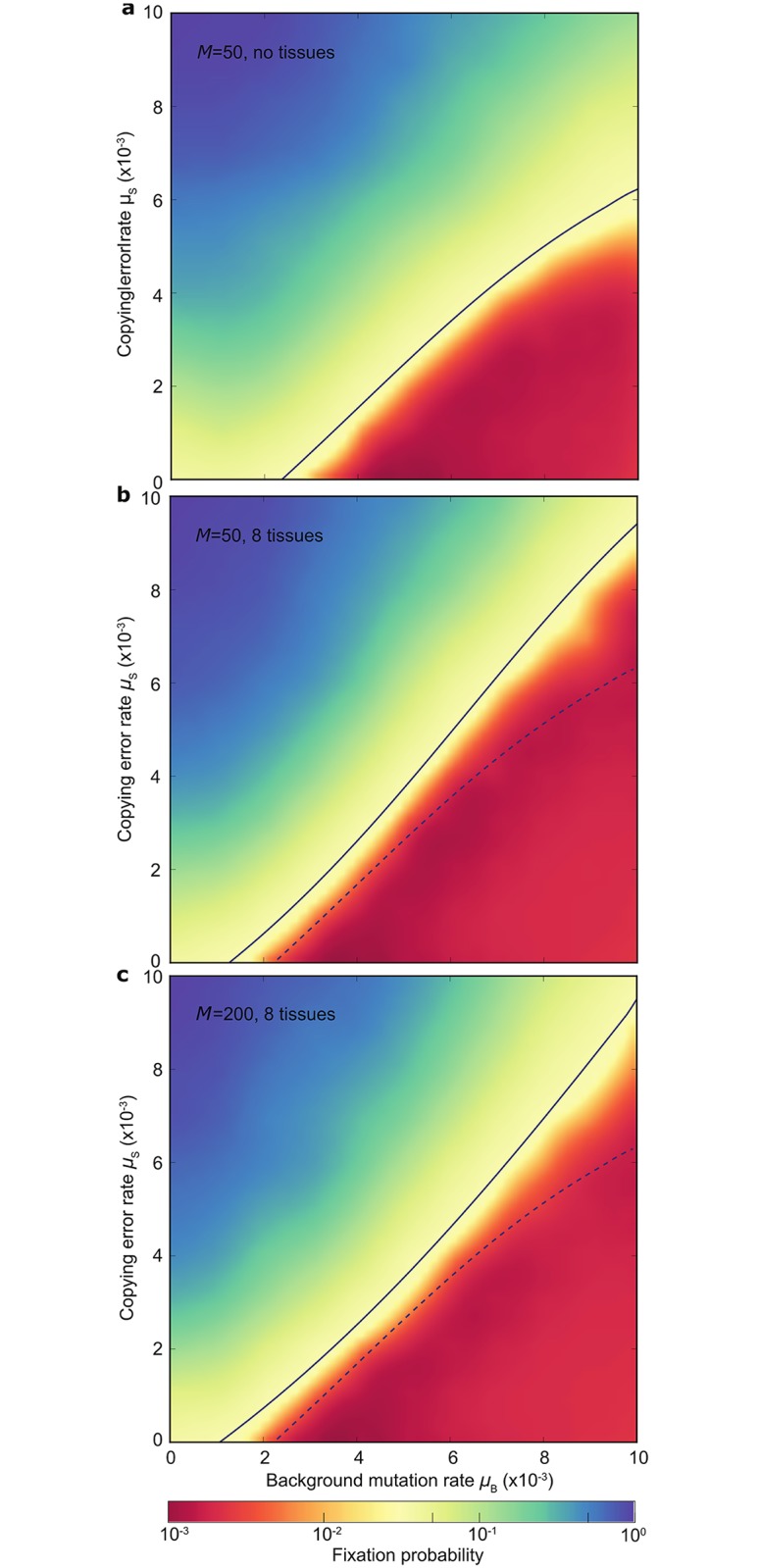
Germline evolution depends on mitochondrial mutation rate. **(a)** Heat map showing fixation probability of an allele encoding early germline sequestration (at generation *N*_*G*_ = 3) in a simple organism that lacks tissue differentiation and produces gametes by somatic gametogenesis (at generation *N*_*G*_ = 10), in relation to the rate of copying errors (*μ*_S_) and background damage (*μ*_B_). The early germline mutation is introduced at a frequency of 0.05 (see Methods). Early germline sequestration is favoured by higher *μ*_S_ and lower *μ*_B_ (blue, top left). The early germline allele is selected against in organisms with low *μ*_S_ and high *μ*_B_ (red, bottom right), conditions that instead favour somatic gametogenesis. The solid line represents neutrality. **(b)** Increasing the number of tissues to eight makes it harder to fix an early germline (*N*_*G*_ = 3)—the region shaded in red expands (solid line versus dotted line) so germline fixation now requires higher *μ*_S_ and lower *μ*_B_ compared with (a). **(c)** Increasing the number of mitochondria to 200 in an organism with eight tissues has little effect on early germline sequestration. Thus, increasing the level of complexity (more tissues and mitochondria) does not favour early germline sequestration. Underlying data can be found at: https://github.com/ArunasRadzvilavicius/GermlineEvolution/tree/master/FigureData.

When mitochondrial mutation input through copying errors is low, the benefit of increased segregational variance between gametes tends to outweigh the benefit of avoiding copying errors (*μ*_S_) through arresting cell division in an early sequestered germline. At low *μ*_S_, more rounds of cell division increase the proportion of gametes with a reduced mutation load despite the higher net input of copying error mutations (Fig 2a and 2b). Germline sequestration is therefore unlikely to evolve in organisms with low *μ*_S_ (Fig 3a). This outcome depends on the input of copying errors relative to the background mutation rate (*μ*_B_). Background mutations occur whether oocytes are sequestered early in development or are derived late from somatic tissues. If background mutations dominate (Fig 3a, bottom right corner), a germline is unlikely to evolve. Only when the copying error rate (*μ*_S_) increases markedly relative to the background mutation input (*μ*_B_) does selection favour an early sequestered germline, with a marked reduction in the number of cell divisions leading to gamete production (Fig 3a).

**Fig 4:**
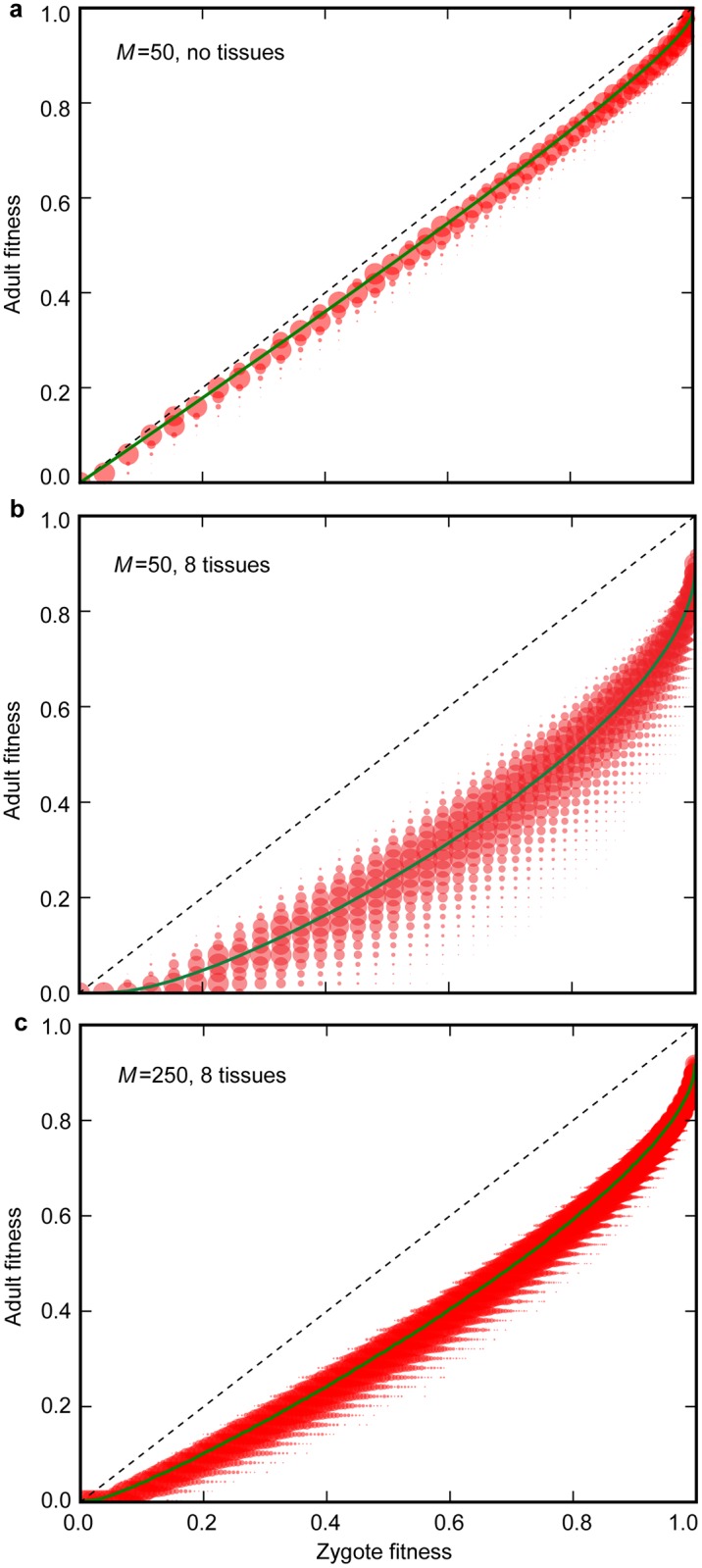
Mitochondrial segregation undermines adult fitness. Adult fitness is a function of zygote fitness (the mutation load inherited), mutational input, and random segregation during development. **(a)** In organisms with no tissue differentiation, adult fitness is similar to zygote fitness, as the number of new mutations accumulating within a single generation is limited, and variance in mutant load between cells within a tissue has no effect on adult fitness. **(b)** In organisms with early differentiation of multiple tissues, adult fitness is undermined by segregational variance, as some tissue-precursor cells receive a higher mutant load than others, and adult fitness depends on the function of the worst tissue. **(c)** Increasing the number of mitochondria decreases the variance in mutant load between tissue-precursor cells, and so reduces the loss in adult fitness caused by random segregation. Parameter values *μ*_S_ = 0.01, *μ*_B_ = 0.005, ten cell divisions to adulthood, and a lifespan equivalent to 40 cell division cycles. Underlying data can be found at: https://github.com/ArunasRadzvilavicius/GermlineEvolution/tree/master/FigureData.

Importantly, increasing the number of tissues (Fig 3b) or mitochondria (Fig 3c), which both correspond to greater complexity, does not enhance the likelihood of germline evolution. The reason again relates to segregation. In organisms with a single tissue, mean adult fitness closely follows the mutation load inherited by the zygote, with some variation induced by mutation accumulation (Fig 4a). In contrast, with multiple tissues, mutations can segregate differentially into distinct tissues, and with strong tissue epistasis (organism fitness depends on the mutational state of the worst tissue) will cause a substantial reduction in mean and increase in variance of adult fitness relative to the inherited zygote mutation load (Fig 4b). This creates heightened selective pressure for the late production of gametes as the extra rounds of segregation creates greater variance in mutation load between gametes and allows a stronger evolutionary response compared to competitors with early germline sequestration (Fig 3b). An early germline is less likely to evolve compared to an organism with a single tissue for the same mutation parameters.

Another marker of increased complexity is an increase in the number of mitochondria (*M*). This has a different effect. With higher *M*, segregation during development causes a more muted decrease in mean and increase in variance of adult fitness (Fig 4c). This is beneficial for adult fitness but has a knock-on effect of reducing fitness variation among gametes and thus weakens the evolutionary response. The net effect on selection for early germline sequestration is minimal (Fig 3c). Overall, then, greater complexity caused by multiple tissues and high numbers of mitochondria does not by itself contribute to the evolution of early germline sequestration, but, if anything, reinforces the stability of somatic gametogenesis.

### Oogamy Improves Adult Fitness and Gamete Quality

Oogamy has typically been interpreted as an outcome of disruptive selection for gamete specialization, with large eggs for provisioning, contrasting with numerous small sperm for success in fertilization [40]. Oogamy is universal in extant metazoan groups and common to multicellular organisms in general, both with and without a dedicated germline, but is most extreme in complex organisms with an early sequestered germline [41]. Our analysis specifies the benefits that flow from the generation of large oocytes with high mitochondrial numbers and the relationship of this to the evolution of a germline.

**Fig 5:**
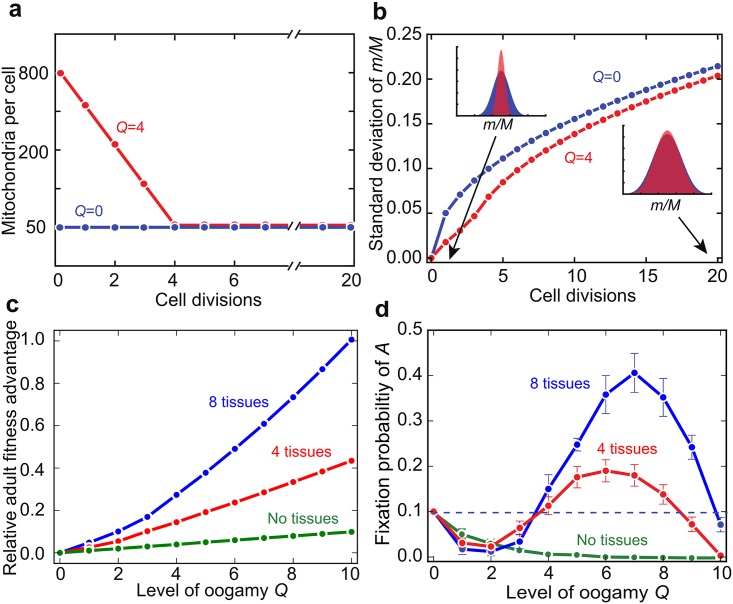
Oogamy improves adult fitness by temporarily suppressing variance. **(a)** The mitochondria in the zygote are partitioned into daughter cells at each cell division. With isogamy (*Q* = 0, blue), the zygote contains the same number *M* mitochondria as normal somatic cells. In contrast, with oogamy (*Q* = 4, red) the larger number of mitochondria contained in the zygotes (2^*Q*^*M* = 800) are partitioned without further replication (over four rounds of cell division) until the standard mitochondrial number (*M* = 50) is restored. **(b)** A large oocyte (*Q* = 4, red) suppresses the variance in mutation load (*m*/*M*) in the first few rounds of cell division (left inset) compared with a small oocyte (*Q* = 0, blue). This early difference in variance is virtually lost after 20 rounds of cell division (right inset). Segregation is modelled as described in the Methods without further accumulation of mutations. *M* = 50 and for illustrative purposes the mutation frequency set at *m* = 25. **(c)** The early reduction in variance produced by oogamy improves adult fitness in organisms with multiple tissues but has practically no effect when there is no tissue differentiation. Mutation rates are set to *μ*_S_ = 0.01 and *μ*_B_ = 0.005, and the number of mitochondria to *M* = 50. The initial mutant load in the zygote is set to 20% (i.e., 2^*Q*^*M*/5). **(d)** The fixation probability (95% confidence intervals) of an allele *A* specifying oogamy *Q* depends on the number of somatic tissues. Increasing *Q* reduces variance in mutant load between tissues, improving somatic fitness (c), but decreases variance among gametes, reducing the efficacy of purifying selection. Moderate levels of mitochondrial oogamy are therefore expected to evolve more readily in organisms with high levels of somatic differentiation. Mutation rates are set to *μ*_S_ = 0.01 and *μ*_B_ = 0.005, and the number of mitochondria to *M* = 50. The dashed line indicates the fixation probability of a neutral mutant. Underlying data can be found at: https://github.com/ArunasRadzvilavicius/GermlineEvolution/tree/master/FigureData.

Oogamy offers a temporary increase in mitochondrial number (*M*) that feeds through to the zygote. Imagine that oocytes undergo *Q* additional rounds of mitochondrial replication without cell division, producing mature eggs and, ultimately, zygotes with 2^*Q*^*M* mitochondria. After fertilization, the first *Q* rounds of cell division partition the initial mitochondrial population in the zygote between daughter cells without the need for further replication, until the baseline *M* mitochondria is restored (Fig 5a). Note that a large oocyte does not alter the mean input of mitochondrial mutations per generation, because replication events lost from early development (when *μ*_*S*_ = 0) are simply moved later into gametogenesis. Nor does it impact the net input of background mutations (*μ*_B_), which is simply a function of lifespan unchanged by oogamy.

The temporary increase in *M* profoundly dampens segregational variance in mutant frequency across the early cell divisions (Fig 5b, left-hand side), which greatly reduces the risk of one tissue inheriting a disproportionate number of mitochondrial mutants. This early suppression of variance is valuable in organisms with multiple tissues (Fig 5c). Once the early divisions of the zygote have allowed cells to regain the standard low level of *M* mitochondria, the many subsequent cell divisions before the end of development allow segregational variation to be established (Fig 5b, right-hand side). So, oogamy readily fixes in organisms with somatic gametogenesis (Fig 5d).

**Fig 6:**
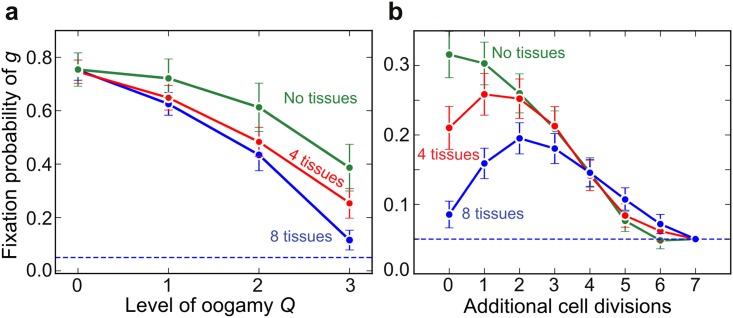
Mitochondrial oogamy opposes early germline sequestration and requires additional germline cell divisions. **(a)** Fixation probability (95% confidence intervals) of the allele *g*, specifying sequestration of an early germline at *N*_*G*_ = 3 cell divisions, decreases with increasing mitochondrial oogamy, especially in organisms with multiple somatic tissues. **(b)** The early germline at *N*_*G*_ = 3 is more likely to fix in organisms with multiple tissues if there are additional germline cell divisions that restore segregational variance between gametes. Parameter values *μ*_S_ = 0.01, *μ*_B_ = 0.005, *M* = 50. The dashed lines indicate the fixation probability of a neutral mutant. Underlying data can be found at: https://github.com/ArunasRadzvilavicius/GermlineEvolution/tree/master/FigureData.

The spread of oogamy under somatic gametogenesis has a strong inhibitory effect on the evolution of a germline. As the level of oogamy (*Q*) increases, the fixation probability of an early germline declines, especially when there are multiple tissues (Fig 6a). Oogamy has the downside that reduced segregation early in development carries over into gametes that are sequestered early in development, and this weakens the response to selection. So an early sequestered germline is less likely to evolve in multi-tissue organisms in which oogamy is already established (e.g., Fig 6a, *Q* = 3).

Yet the observation is that females in active bilaterians invariably combine early germline sequestration with large oocytes, in the extreme packed with several orders of magnitude more mitochondria than are present in normal cells (10^6^ versus ~10^3^) [19]. The model seems at odds with these observations. A solution to this paradox is suggested by the finding that the fixation probability of an early germline rises with additional cell divisions of primordial female germ cells in organisms with multiple tissues (Fig 6b). The extra rounds of cell division in the germline help to restore segregational variance among female gametes, overcoming the suppression of variation caused by oogamy (Fig 6b). There is a balance between restricting *μ*_S_ through early germline sequestration, decreasing variance between tissue precursor cells through oogamy and increasing variance in female gametes through the proliferation of oogonia after germline sequestration. So increasing *Q* in effect favours higher numbers of cell divisions in an early sequestered germline. It is interesting to note that the need for segregational variance in the germline may produce more primordial germ cells than needed and so be accompanied by atresia, the random destruction of female gametes seen across a vast phylogenetic range of bilaterian metazoa [42, 43].

## Discussion

We have considered the evolution of the germline in terms of purifying selection against defective mitochondria. Mitochondrial quality is undermined by mutations, whereas selection is facilitated by increased variance generated through random segregational drift of mitochondria at each round of mitotic division (Fig 1). The more rounds of cell division, the greater the mutational burden (as the mitochondrial population is doubled at each cell division) but the greater the segregational variance, hence visibility to selection at the organism level (Fig 2). In terms of selection for mitochondrial quality, the switch to an early germline with a reduction in the number of germline cell divisions depends mainly on mutations due to copying errors (*μ*_S_) relative to the background mutation rate (*μ*_B_). The balance of these factors explains simply and beautifully why groups with low copying error rates (*μ*_S_), such as plants [44–46] and basal metazoans [47–49], do not sequester a germline; segregational variance is maximised by forming gametes late from the same stem cell population that gives rise to somatic cells (Fig 2). This provides a better opportunity for selection on adult organisms to reduce the mutation load through multiple generations (Fig 3).

Conversely, in groups with higher rates of copying errors (*μ*_S_), mutations accumulate faster during each round of mitotic division, making late differentiation of gametes a liability. This leads to benefit flowing from early sequestration of a dedicated female germ cell lineage with a reduced number of germline cell replication cycles, thereby limiting the accumulation of mutations even though this is at the cost of restricting variance. Examples are bilaterians and ctenophores; both groups have high nucleotide substitution rates in their mitochondria [50–54] and apparently evolved germlines independently [55]. Because mitochondria are not generally transmitted via the male germline, these constraints do not apply to sperm, which can be produced in abundance throughout life. So the simple balance of mutation and segregation explains why somatic gametogenesis is stable and widespread across plants and sessile metazoans, whereas more active metazoans evolved early germline sequestration.

The model presented here is an abstract simplification, and the values used for mutation rates, segregational variance, mitochondrial numbers, and the life cycle are not intended to be realistic (see Methods). Our objective was to examine the general principles involved without getting too bogged down in the detail. Parameter values were chosen for computational expediency. In particular, the mutation rates adopted are considerably higher than seen in most natural systems, and the lifecycle was chosen to be much more simple and condensed. This approach does not affect the general conclusions we draw (see S3 Fig and discussion below). There is considerable uncertainty over the actual range of values for most of the parameters we discuss. For example, mitochondrial mutation rates are normally estimated indirectly from nucleotide substitution rates, which tend to be 10–50-fold higher than the nuclear mean in mammals [50–54], and at least 10-fold lower than the nuclear mean in plants [44–46] and basal metazoans [47–49], a range of about three orders of magnitude. Given that purifying selection over generations eliminates a proportion of mitochondrial mutations, the underlying mutation rates are likely to be higher still. The number of mitochondria per cell and per gamete also vary over many orders of magnitude, from 10^2^ [56–58] to 10^9^ [59–61]. While doubts can be raised about estimates over such a broad phylogenetic range, it seems reasonable to assume that the broad features hold true—that the striking differences in rates of sequence divergence and mitochondrial numbers between plants and basal metazoans and bilaterians do reflect real differences in underlying mutation rates and metabolic demands. We believe these wide natural ranges justify our consideration of the consequence of increasing the error rate associated with mitochondria replication (*μ*_S_) or increasing the number of mitochondria per cell and per oocyte.

We also simplify the life cycle so that early germline sequestration (and tissue segregation) takes place after just a few rounds of cell division, and somatic gametogenesis after 10 rounds of division. In fact, in humans, the oocyte precursor cells undergo about 30 mitotic divisions before entering meiosis [13,62], while the mitotically active cells in the gut [63] or blood [64] may undergo hundreds or even thousands of rounds of cell division. Likewise, somatic gametogenesis in mature sponges, corals, and large land plants may involve considerable rounds of mitotic division [65,66]. By modelling a much larger and perhaps more realistic life cycle with 60 cell divisions, we observe conditions favouring an early germline (set in this case at *N*_*G*_ = 10) with a minimal mutation rate approximately two orders of magnitude lower than in Fig 3, without altering the relative contribution of each parameter (S3 Fig). We present these data to reinforce the point that our modelling is best interpreted in light of the relative strength of the parameters involved, not the absolute values. Relatively high mutation rates will tend to drive germline evolution; relatively low mutation rates produce higher quality gametes when coupled with the greater segregational variance generated by somatic gametogenesis.

An important relative difference is the balance between copying errors (*μ*_S_) and background mutations (*μ*_B_) (Fig 3). Historically, most mitochondrial mutations were assumed to be caused largely by ROS leak from active mitochondria [67–70], and to a lesser extent by other forms of oxidative stress such as UV radiation [71]. Mitochondrial DNA was claimed to be naked (lacking histones [72]), inadequately repaired [72], and vulnerable to ROS damage from the adjacent respiratory chains [67–70]. More recent work suggests that this one-sided view is far from correct [24–27,72–77]. Mitochondrial DNA is protected by DNA-binding proteins, notably mitochondrial transcription factor A [73]; most of the DNA repair mechanisms that operate in the nucleus have mitochondrial equivalents [74, 75], and the most common ROS, such as superoxide, cause little oxidative damage to mitochondrial DNA [76], acting instead as critical signals in regulatory pathways [77]. In contrast, work with *polG* mutants in mice suggests that the majority of mitochondrial mutations are copying errors [24–27]. More broadly, the strong bias towards transition mutations in the mitochondrial DNA of bilaterians is thought to be associated with copying errors rather than ROS damage and UV radiation [24,76,78]. But these observations do not rule out a significant contribution of background mutations, as implied by a modest proportion of transversion mutations in mammals [24,76], which tend to be associated with oxidative damage rather than copying errors [24,76,78].

We have accordingly taken both copying errors (*μ*_S_) and background mutations (*μ*_B_) to be significant contributors to the mitochondrial mutation rate, but with varying contributions depending on lifestyle and phylogeny. The model suggests that somatic gametogenesis is stabilized by relatively high *μ*_B_ (Fig 3). If a high proportion of transversion mutations are indeed associated with oxidative damage [24,76,78], then this fits well with the observations. Unlike most animal mitochondrial genomes, basal metazoans including sponges, corals, and placozoans all have very low *μ*_S_ [47–49] but a relatively high proportion of transversion mutations [48], equating roughly to a high *μ*_B_ in our model. This combination of low *μ*_S_ with high *μ*_B_ readily explains why these major metazoan phyla lack a germline. Similarly, most plants have low mutation rates (10–20-fold lower than in their nuclear genomes [44–46]) and typically display no bias towards transition mutations in mitochondrial DNA [46]. This again suggests a relatively high proportion of mutations caused by oxidative damage relative to copying errors, equating to relatively high *μ*_B_. Whether these mutations are produced by ROS leak or by relatively high exposure to UV radiation (given phototropic environments, long lifespans and exposed gamete precursor cells not shielded in ovarian tissue) is not clear. Nonetheless, our predictions are clear: organisms with relatively low *μ*_S_ and high *μ*_B_ will tend to favour somatic gametogenesis (Fig 3), exactly as happens in plants and basal metazoans that lack an early sequestered germline. We are not aware of any other hypothesis that explains so cleanly why these large groups never developed germline sequestration, despite their elaborate tissue differentiation and morphological complexity.

Why did the copying-error rate (*μ*_S_) increase in the lineages giving rise to bilaterians and ctenophores? The rise could have been linked with an ecological shift from sessile filter feeding in the Ediacaran to motility and predation in the Cambrian. More aerobic activity is linked with more protein synthesis and tissue turnover, so more replication of mitochondrial DNA between each cell division is needed to meet metabolic demands [79–81]. Increasing oxygen levels in the late Neoproterozoic [82] enabled multiple trophic levels and predation for the first time, as energy conservation from aerobic respiration approaches 40% compared with <10% from fermentation, making predation virtually impossible in anoxic worlds [83,84]. In the early Cambrian, predation is thought to have driven an evolutionary arms race, leading to greater size and more physical activity in bilaterians and ctenophores [85]. The evolution of a germline in turn frees mitochondria in somatic tissues from metabolic constraints, enabling greater power and tissue differentiation. A link between mitochondrial mutation rate and germline evolution is corroborated by the Ceriantharia, which, unlike other Anthozoa, have fast-evolving mitochondrial DNA [86], suggesting a relatively high *μ*_S_; strikingly, their larvae have gonads and are apparently paedogenetic [87], implying that germline sequestration may have evolved in this group. Conversely, the secondary evolution of somatic gametogenesis in other groups such as Ectoprocta and Entoprocta [88] could have been favoured by their sessile lifestyles coupled to a falling *μ*_S_. Little is known about mitochondrial sequence divergence in these groups, but we predict their *μ*_S_ will be lower than in related metazoans that retain a germline.

An unexpected finding of the model was that greater complexity, measured as the number of tissues, did not enhance selection for a germline. While segregation is helpful in generating variance between gametes, it potentially has a detrimental effect early in development, as it can result in the biased accumulation of mutants in the progenitor cells of particular tissues. Some tissue progenitors will contain more mutant mitochondria than others, and this will depress organismal fitness, given tissue epistasis (Fig 4). That is certainly true of bilaterians, in which fitness is dependent on the mutual functioning of all tissues; for example, brain function depends on heart and lung function for proper oxygen supply. Two effects are relevant here. First, as somatic gametogenesis generates greater variance between gametes and produces a stronger selective reduction in mutation load, it is more strongly favoured with multiple tissues (Fig 3b). This retards the evolution of an early germline. Second, multiple tissues favour the evolution of oogamy. Increasing the number of mitochondria in oocytes (high *Q*) means that early cell division occurs with a much-reduced effect of segregation, as drift is suppressed by large numbers (Fig 5b). This causes a reduction in mutational variation between cells in early development, thereby improving adult fitness when there are multiple tissues (Fig 5c). But this again retards the evolution of an early germline, because multiple cells divisions are required after the reestablishment of the normal level of *M* mitochondria per cell to generate a reasonable level of variance between gametes (Fig 6a).

Strikingly, some plants and basal metazoans have smaller numbers of mitochondria in oocytes (i.e., lower values of *Q*) than are typical of bilaterians [56–58], suggesting that “mitochondrial oogamy” is distinct from the large size of oocytes for provisioning. This lower degree of mitochondrial oogamy can be explained by the lower mutual dependence of adult fitness on organ function in plants and basal metazoans (i.e., less epistasis between tissue fitness). In the model, this is equivalent to a smaller number of tissues, and we find that oogamy is less strongly favoured with fewer tissues (Fig 5d compares four and eight tissues). Nonetheless, even in plants there is a degree of embryonic tissue determination in early development (e.g., in seeds [66]), so moderate mitochondrial oogamy is predicted.

The model reveals that high mutation rates (*μ*_S_) favour early germline sequestration, whereas tissue differentiation early in development favours large oocytes packed with mitochondria, and this retards the evolution of an early germline. The problem is that, when large oocytes are combined with early germline sequestration, the variance between oocytes is minimized, hindering selection. We suggest that this may help explain follicular atresia, a long-puzzling feature of germline physiology. In human development, >6 million oogonia are generated by mitotic proliferation during foetal development, followed by the seemingly random apoptotic death of all but 500,000 of them (i.e., >90%) by the start of puberty [42]. A similar pattern is common across most bilaterian metazoans [43], including nematodes such as *Caenorhabditis elegans*, in which >50% of oocytes die [89]. Some kind of selective advantage for atresia has long been sought, largely with the idea of oocyte quality control [90]. But no plausible mechanism has been identified, and it is hard to imagine that >90% of oogonia have low fitness. Seen in terms of maximising variance, however, atresia makes much more sense. The proliferation of oogonia necessarily generates segregational variance between them, some of which now have the chance of carrying few or no mitochondrial mutations (Fig 2). Proliferation generates far more oogonia than are needed for fertilization. In the model, allowing additional rounds of segregation within the germline does indeed promote early germline fixation (Fig 6b). In real life, this “over supply” is rectified by random elimination of nine out of every ten oogonia, with the survivors being “islands” of segregational variance, more different to each other than would be the case if fewer divisions were used to generate an equal number of oocytes. Functional selection could now more realistically take place, for example, in the competition between follicles during oocyte maturation. This may partially account for the reported germline selection against severe mitochondrial mutations in mice [91–93].

Segregation is not the only way to generate variance between oocytes. The idea that has garnered the most empirical [94–96] and theoretical [36,37,97] attention is the so-called mitochondrial germline bottleneck. It is commonly asserted that mitochondrial populations are reduced to low numbers at a single stage of germline development, and some evidence supports this, albeit the low number attained is contested [98]. However, there is rather little evidence that this is anything other than the reduction from the high content of the oocyte back to a normal level. As we have shown, segregation per se has a similar effect, so the need for a bottleneck may have been overstated. Nonetheless, models suggest that, in principle, a very low *M* at some point during germline development would increase variance between oocytes, possibly limiting the need for proliferation of oogonia. Another possibility is that only a subset of oocyte mitochondria is replicated during oocyte maturation [99], which, again, would increase the variance between oocytes. It would not be surprising if different species had evolved different mechanisms for generating variance between oocytes, or that the need for variance differs amongst groups with divergent ecologies and lifestyles. The concentration on the details of a very small subset of model organisms detracts from understanding how evolution has acted in different ways to related problems.

The rationale we have put forward for the evolution of the germline is that it minimises the accumulation of mutations due to copying errors in mitochondrial replication (*μ*_S_). The total accumulation of new mutations will also be lowered by adaptations that suppress background damage (*μ*_B_) in female germ cells. A number of features appear to fulfil this role: protecting oocytes in an internal ovary, provisioning them from follicular cells, and repressing the transcription of mitochondrial genes and active respiration to reduce exposure to ROS, giving rise to quiescent oocytes [15,18–20]. The model shows that the early sequestration of large oocytes is more likely to be favoured when accompanied by specific lowering of *μ*_B_ (Fig 3 and S4 Fig). This is the case in the female germline of most [15,19] but not all [28,29] bilaterians. Even when mitochondria are active it is possible that other specific adaptations could suppress *μ*_B_, for example, through upregulating antioxidant or DNA repair enzymes [28]. From our point of view, all these features are secondary adaptations that have arisen after the germline has become established. Likewise, once a germline is established, there is no longer a constraint on the replication error rate (*μ*_S_) among somatic cell lineages. These are now free to become “disposable,” as their lineage will no longer contribute to the heritable germ material.

### Conclusions

For the last 30 years, the dominant explanation for the evolution of early germline sequestration has been that it was essential for the emergence of multicellular organisms through the suppression of selfish conflict between the cells that make up an individual. At its heart, this viewpoint lacks a rationale for the evolutionary stability of numerous organisms that lack a germline.

Here we have turned this paradigm on its head, locating the key driving force in selection against mitochondrial mutations. This reflects the interplay of mutation and segregation of mitochondrial mutations in gametes and tissues. With low copying-error rates (*μ*_S_), the combination of segregation and selection improves mitochondrial quality over generations, favouring somatic gametogenesis in plants and sessile metazoans. Higher *μ*_S_ drives early germline sequestration, which ultimately permits greater tissue differentiation and complexity in groups such as bilaterians and ctenophores. The increase in *μ*_S_ probably related to the rise in oxygen shortly before the Cambrian explosion [82]. We find that the evolution of complex development, with stronger epistasis between tissues, requires suppression of mitochondrial variance between tissue-progenitor cells. This is achieved by large oocytes containing high mitochondrial numbers. That, in turn, risks the loss of segregational variance between oocytes sequestered in the germline early in development. The solution was to restore segregational variance through the mitotic proliferation of oogonia in the germline, followed by atresia, maximizing the variance between the surviving oocytes.

These findings set out a completely novel hypothesis for the origin and evolution of germline sequestration, which predicts the traits of groups from plants and sponges to ctenophores and bilaterians. Unlike other hypotheses, selection for mitochondrial quality can account for the stability of both somatic gametogenesis and early germline sequestration. The requirement for segregational variation in the germline also elucidates the potential risks associated with mitochondrial heteroplasmy, which reduces variance between oocytes while increasing the risk of segregating mitochondrial mutants into particular tissues, lowering adult fitness and contributing to mitochondrial disease. The unusual population genetics of mitochondrial mutation and segregation can uniquely explain the evolution of the germline in complex bilaterians.

## Methods

### Segregational Drift Generates Variance in Mitochondrial Mutation Load

Here we review the mitochondrial dynamics at the cell level, showing that random segregation at every cell division generates variation in the mutational load (Fig 2). We start with a cell containing *M* mitochondria, out of which *m*_0_ are mutant. The initial state of an infinite population is then represented by a state vector **p**^(0)^, with all of its *M*+1 entries set to zero, except for the *m*_0_-th, which is set to one, i.e., $p_{i}^{\left(0\right)}=\delta \left(i,m_{0}\right)$. A single cell cycle consists of mitochondrial mutation, replication, and cell division. Mitochondrial mutation is a Bernoulli trial with success probability *μ*, while segregation is modelled as a simple random sampling without replacement. After *n* cell divisions, the population state vector becomes

$$p^{\left(n\right)}={\left(KJ\right)}^{n}p^{\left(0\right)}.$$

Here, **J** is the (*M* + 1) × (*M* + 1) matrix with elements representing the transition probabilities due to mutation,

$$J_{q,m}=p_{mut}\left(q;m,M\right)=\left(\begin{array}{c} M-m\\ q-m \end{array}\right)\mu^{q-m}{\left(1-\mu \right)}^{M-q}.$$

Similarly, **K** is the matrix of same dimensions representing the transformation due to random sampling,

$$K_{q,m}=p_{seg}\left(q;m,M\right)=\frac{\left(\begin{array}{c} 2m\\ k \end{array}\right)\left(\begin{array}{c} 2M-2m\\ M-k \end{array}\right)}{\left(\begin{array}{c} 2M\\ M \end{array}\right)}.$$

In Fig 2, we show distributions **p**^(*n*)^ for *n* = 0…10.

Variance in the mutant load after *n* cell divisions can be expressed analytically in the illustrative case of *μ* = 0, where only segregational drift is accounted for. Let *X*_*n*_ be a random variable denoting the number of mutant mitochondria within a cell in the developing tissue or embryo after *n* cell divisions, and *x*_*n*_ its actual realization. For sampling from the hypergeometric probability distribution, the population mean equals the initial number of mutants, E(*X*_*n*_) = *x*_0_, while the variance can be decomposed as

$$\text{Var}\left(X_{n}\right)=\text{E}\left[\text{Var}\left(X_{n}|X_{n-1}\right)\right]+\text{Var}\left[\text{E}\left(X_{n}|X_{n-1}\right)\right].$$

For sampling without replacement from the hypergeometric probability distribution, the conditional variance is

$$\text{Var}\left(X_{n}|x_{n-1}\right)=\frac{x_{n-1}\left(M-x_{n-1}\right)}{2M-1},$$

and so

$$\begin{array}{l} \text{Var}\left(X_{n}\right)=\text{E}\left(\frac{X_{n-1}\left(M-X_{n-1}\right)}{2M-1}\right)+\text{Var}\left(X_{n-1}\right)\\ =\text{E}\left(\frac{MX_{n-1}}{2M-1}\right)-\text{E}\left(\frac{X_{n-1}^{2}}{2M-1}\right)+\text{Var}\left(X_{n-1}\right)\\ =\frac{M}{2M-1}\text{E}\left(X_{n-1}\right)-\frac{1}{2M-1}\text{E}\left(X_{n-1}^{2}\right)+\text{Var}\left(X_{n-1}\right)\\ =\frac{x_{0}M}{2M-1}-\frac{1}{2M-1}\left[\text{Var}\left(X_{n-1}\right)+x_{0}^{2}\right]+\text{Var}\left(X_{n-1}\right)\\ =\frac{x_{0}\left(M-x_{0}\right)}{2M-1}+\left(1-\frac{1}{2M-1}\right)\text{Var}\left(X_{n-1}\right). \end{array}$$

Here, *x*_0_ is the initial number of mutants within a cell. This is a recurrence relation of the form *h*_*n*_ = *Hh*_*n*−1_ with *h*_*n*_ = *Var*(*X*_*n*_) − *x*_0_(*M* − *x*_0_) and $H=1-\frac{1}{2M-1}$. With the boundary condition Var(*X*_0_) = 0, *h*_0_ = −*x*_0_(*M* − *x*_0_), the solution is

$$\text{Var}\left(X_{n}\right)=x_{0}\left(M-x_{0}\right)\left[1-{\left(1-\frac{1}{2M-1}\right)}^{n}\right].$$

Variance in the mutant frequency $P_{n}=\frac{X_{n}}{M}$ is then

$$\text{Var}(P_{n})=p_{0}(1-p_{0})\left[1-{\left(1-\frac{1}{2M-1}\right)}^{n}\right].$$

In oogamous matings, the zygote contains 2^*Q*^ *M* mitochondria and undergoes *Q* divisions without mitochondrial replication. Changes in variance follow a similar trend, but this time the recurrence relation is

$$\begin{array}{cc} \text{Var}(P_{n})=\frac{p_{0}(1-p_{0})}{2^{1+Q-n}M-1}+Var(P_{n-1})(1-\frac{1}{2^{1+Q-n}M-1}), & 1\leq n\leq Q. \end{array}$$

The recurrence relation was used directly to produce Fig 5b. For a large initial number of mitochondria, $\frac{1}{2^{Q}M}\to 0$ and the approximate solution is

$$\text{Var}\left(P_{n}\right)=\frac{p_{0}\left(1-p_{0}\right)}{2^{Q}M}\left(2^{n}-1\right),\;\;1\leq n\leq Q.$$

### Finite-Population Simulation Model for the Evolution of Germline

To study the evolutionary dynamics of alleles responsible for germline sequestration, we developed a multilevel, agent-based model, implemented as a set of simulation routines in ANSI/ISO C++ (https://github.com/ArunasRadzvilavicius/GermlineEvolution). The model population consists of *N* = 500 multicellular individuals, with equal numbers of males and females. A single individual is represented as an object containing 2^*L*^ cells, each containing a number of mutant and wild-type mitochondria (that is, development consists of *L* cell divisions). The cells are grouped into *X* tissues, each of which is composed of 2^*L*^/*X* cells. Tissues are differentiated after *T* cell divisions by assigning each precursor cell into its own tissue, so that *X* = 2^*T*^. We measure time in terms of cell divisions, with a total life span before gamete production and release *S* = 40. Generations are discrete and non-overlapping. Organism development proceeds by iterating through the population and modifying these objects according to a set of predefined rules. The population is initialized in a random mutational state, and the simulation proceeds as follows:

1. Start the generation with a population of *N* zygotes;
2. For each organism in the population, repeat until development is complete after *L* divisions:
  - For every somatic cell within an organism:
    - Apply background mitochondrial mutation as a Bernoulli event with the success probability *μ*_*B*_. In practice, this step is implemented by drawing a single random number from the binomial probability distribution Binom(*M* − *m*, *μ*_*B*_), where *m* is the initial number of mitochondrial mutants.
    - If the number of mitochondria is *M*, apply mitochondrial mutation as a singular Bernoulli trial with success probability *μ*_*S*_ (a number of new mutants is drawn from Binom(*M* − *m*, *μ*_*S*_)), then duplicate the mitochondrial population.
    - Partition mitochondria into two daughter cells by sampling without replacement, which in practice is implemented by drawing a random number form the hypergeometric probability distribution Hypergeom(2*M*, 2*m*, *M*).
  - After *N*_*G*_ cell divisions, set aside a single primordial germ cell by copying a randomly chosen cell, which does not undergo further mitotic divisions.
  - After *T* cell divisions, assign 2^*T*^/*X* cells to each tissue.
  - Development (i.e., cell division) stops after *L* cell divisions.
  - After development has stopped, continue to apply background mitochondrial mutation to all cells until *S* time units, each time with probability *μ*_*B*_. This applies both to primordial germ and somatic cells.
3. Apply selection by sampling *N*/2 males and *N*/2 females from the population with replacement, linearly weighted according to their mitochondrial fitness.
4. In female germ cells, apply mitochondrial mutation as a binomial event with probability *μ*_*S*_, then duplicate the mitochondrial population. Do this *Q* times to account for mitochondrial oogamy
5. Complete gametogenesis by executing two meiotic cell divisions, with mitochondrial sampling without replacement; male gametes contain *M*/2 and eggs contain 2^*Q*^ *M*/2 mitochondria.
6. Fuse random gamete pairs of opposite sexes assuming uniparental inheritance of mitochondria, with resampling after fusion to bring the zygote content to 2^*Q*^ *M* mitochondria.

### Evolution of the Nuclear Alleles and Fixation Statistics

Mating type, the number of germline cell divisions *G*, the log-size of the zygote *Q*, and the degree of uniparental inheritance *v* are all traits of an organism controlled by a set of loci in the nuclear genome, which is assumed to be diploid. The mating type locus is heterogametic *ZW* in females, while the other mating types is homogametic *ZZ*.

We determine the selective advantage of an invading allele in a Monte Carlo simulation, by numerically calculating its fixation probability within a finite population. With the population at equilibrium, the invading allele is introduced at a low frequency *f*_0_ = 0.05 and its fate is tracked until either fixation or extinction. This requires 10^3^ − 10^4^ repetitions of the calculation, depending on the mutation rates and the associated levels of noise. This pattern is assessed relative to the fixation probability of a neutral allele, which simply equals its initial frequency *f*_0_. The error bars in Figs 5 and 6 represent the 95% confidence interval of the binomial proportion via the Gaussian approximation, that is $\Delta p=\pm 1.96\sqrt{\frac{p\left(1-p\right)}{n}}$, where *n* is the number of trials and *p* is the measured rate of fixation. An allele is deemed to be evolutionarily advantageous if its fixation probability exceeds the chance of fixation of the neutral allele. The model parameters, such as mutation rate and the strength of epistatic interactions, are not intended to correspond to values measured in specific species, but to show general patterns and to maintain reasonable calculation times (unavoidable in modelling studies).

The same general procedure is applied to determine the fate of modifiers coding for the number of germline cell divisions, the level of mitochondrial oogamy, or mitochondrial exclusion. The germ cell differentiation locus is expressed in both sexes and is assumed to be autosomal, with the invading allele assumed to be dominant. We also examined other dominance states, but the main conclusions of this work remained unaffected. The other two loci are *W* linked and unaffected by dominance considerations.

## Supporting Information

S1 Fig Fixation of early germline segregation under varying mitochondrial fitness functions. Early germline sequestration (at generation *N*_*G*_ = 3) evolves under high mitochondrial DNA copying error rates (*μ*_S_), regardless of the shape of the fitness function *ω*(*m*) = 1—*s*(*m*/*M*)^*ξ*^, where *ξ* measures the shape of the fitness function: concave (*ξ = 2*), linear (*ξ* = 1) or convex (*ξ* = 0.5). The number of mitochondria per cell is set to *M* = 50, with selection strength *s* = 1. The dashed line indicates the fixation probability of a neutral mutant. Underlying data can be found at: https://github.com/ArunasRadzvilavicius/GermlineEvolution/tree/master/FigureData.
(PNG)

S2 Fig Evolution of germline sequestration under weaker selection against mutants. The evolution of early germline sequestration (at generation *N*_*G*_ = 3) is favoured by high copying error mutation rates (*μ*_S_). In finite populations, the absolute fixation probability of the germline allele is lower under weaker selection *s* against mutants as in Eq 1, because this increases the relative importance of random drift. The threshold mutation rate (*μ*_S_) at which an early germline becomes favourable, however, is not greatly affected. The dashed line indicates the fixation probability of a neutral mutant. Underlying data can be found at: https://github.com/ArunasRadzvilavicius/GermlineEvolution/tree/master/FigureData.
(PNG)

S3 Fig Germline evolution in an organism with an extended lifecycle. Fixation probability of an allele encoding early germline sequestration (at generation *N*_*G*_ = 10) in an organism with an extended life cycle that produces gametes by somatic gametogenesis at generation *N*_*G*_ = 60. The number of somatic cell divisions is significantly higher than in the simulations used in the main text where we considered invasion of an early germline at generation *N*_*G*_ = 3 for an organism with a life cycle of *N*_*G*_ = 10 generations. With the extended life cycle there is a much higher opportunity for the build-up of segregational variation and as a result early germline sequestration is favoured with mitochondrial mutation rates that are ~2 orders of magnitude lower. Once again, early germline sequestration is favoured by low background mutation rates (*μ*_B_) and high replication error rates (*μ*_S_). To reduce computational complexity, the adult fitness of an organism has been approximated according to the probability distribution function **p**^(60)^ as defined in the Methods, while the number of mutant mitochondria in gametes is drawn from probability distribution functions **p**^(60)^ and **p**^(10)^. The number of mitochondria per cell is *M* = 50, selection strength *s* = 1, with a single tissue. The dashed line indicates the fixation probability of a neutral mutant. Underlying data can be found at: https://github.com/ArunasRadzvilavicius/GermlineEvolution/tree/master/FigureData.
(PNG)

S4 Fig Restricting background mutations favours germline fixation. The evolution of early germline sequestration (at generation *N*_*G*_ = 3) and oogamy (*Q* = 3) is favoured by low background mutation rates (*μ*_B_). The allele *A* specifying early germline sequestration is more likely to be fixed at higher values of mutation due to copying errors (*μ*_S_). Germline fixation also depends on the background mutation rate (*μ*_B_). Germline sequestration does not fix if *μ*_B_ > 6 x 10^−3^ but fixes readily at lower *μ*_B_, even when *μ*_S_ is low. Adaptations that favour low *μ*_B_ are characteristic of bilaterians germlines, including sequestration of oocytes in an internal ovary, repression of transcription and translation, and suppression of mitochondrial respiration [17,21]. The dashed line indicates the fixation probability of a neutral mutant. Underlying data can be found at: https://github.com/ArunasRadzvilavicius/GermlineEvolution/tree/master/FigureData.
(PNG)

## Abbreviations

ROS: reactive oxygen species
UV: ultraviolet
