# Supplementary figures and images for: Selection for Mitochondrial Quality Drives Evolution of the Germline
Source: PLoS Biol. 2016 Dec 20;14(12):e2000410. doi: 10.1371/journal.pbio.2000410 (PMC5172535; doi:10.1371/journal.pbio.2000410)

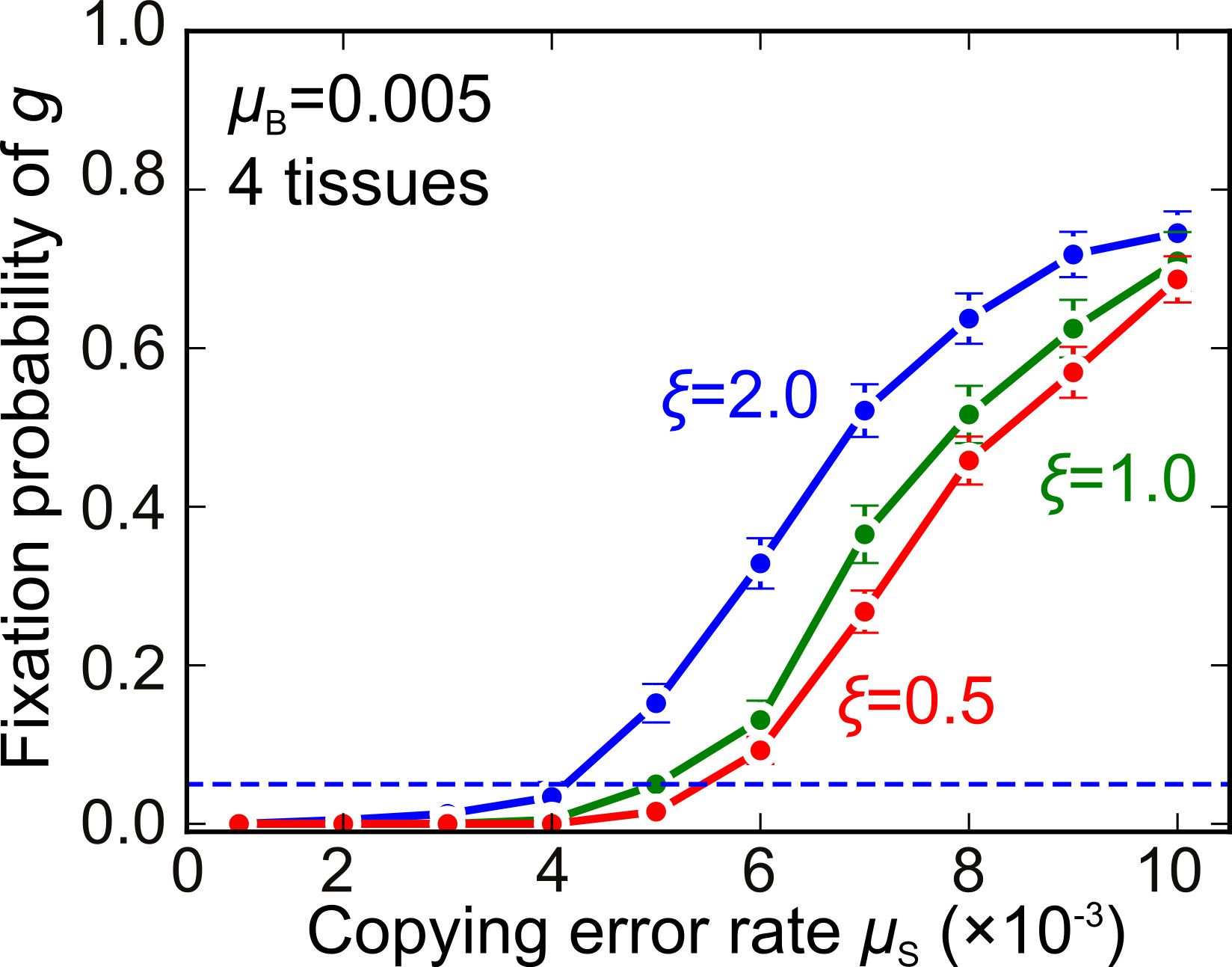

Supplement: S1 Fig — Early germline sequestration (at generation NG = 3) evolves under high mitochondrial DNA copying error rates (μS), regardless of the shape of the fitness function ω(m) = 1—s(m/M)ξ, where ξ measures the shape of the fitness function: concave (ξ = 2), linear (ξ = 1) or convex (ξ = 0.5). The number of mitochondria per cell is set to M = 50, with selection strength s = 1. The dashed line indicates the fixation probability of a neutral mutant. Underlying data can be found at: https://github.com/ArunasRadzvilavicius/GermlineEvolution/tree/master/FigureData. (PNG) [file pbio.2000410.s001.png]

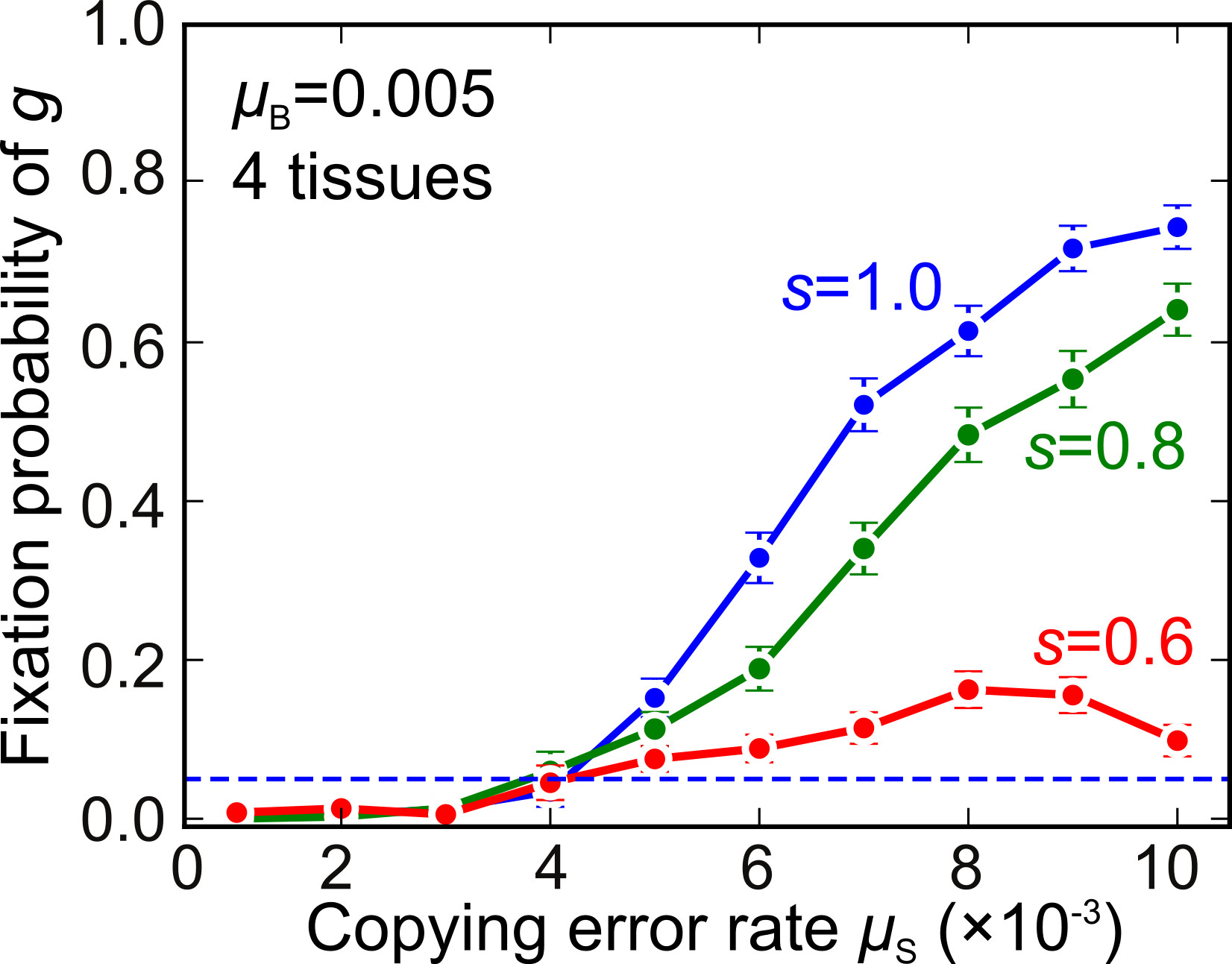

Supplement: S2 Fig — The evolution of early germline sequestration (at generation NG = 3) is favoured by high copying error mutation rates (μS). In finite populations, the absolute fixation probability of the germline allele is lower under weaker selection s against mutants as in Eq 1, because this increases the relative importance of random drift. The threshold mutation rate (μS) at which an early germline becomes favourable, however, is not greatly affected. The dashed line indicates the fixation probability of a neutral mutant. Underlying data can be found at: https://github.com/ArunasRadzvilavicius/GermlineEvolution/tree/master/FigureData. (PNG) [file pbio.2000410.s002.png]

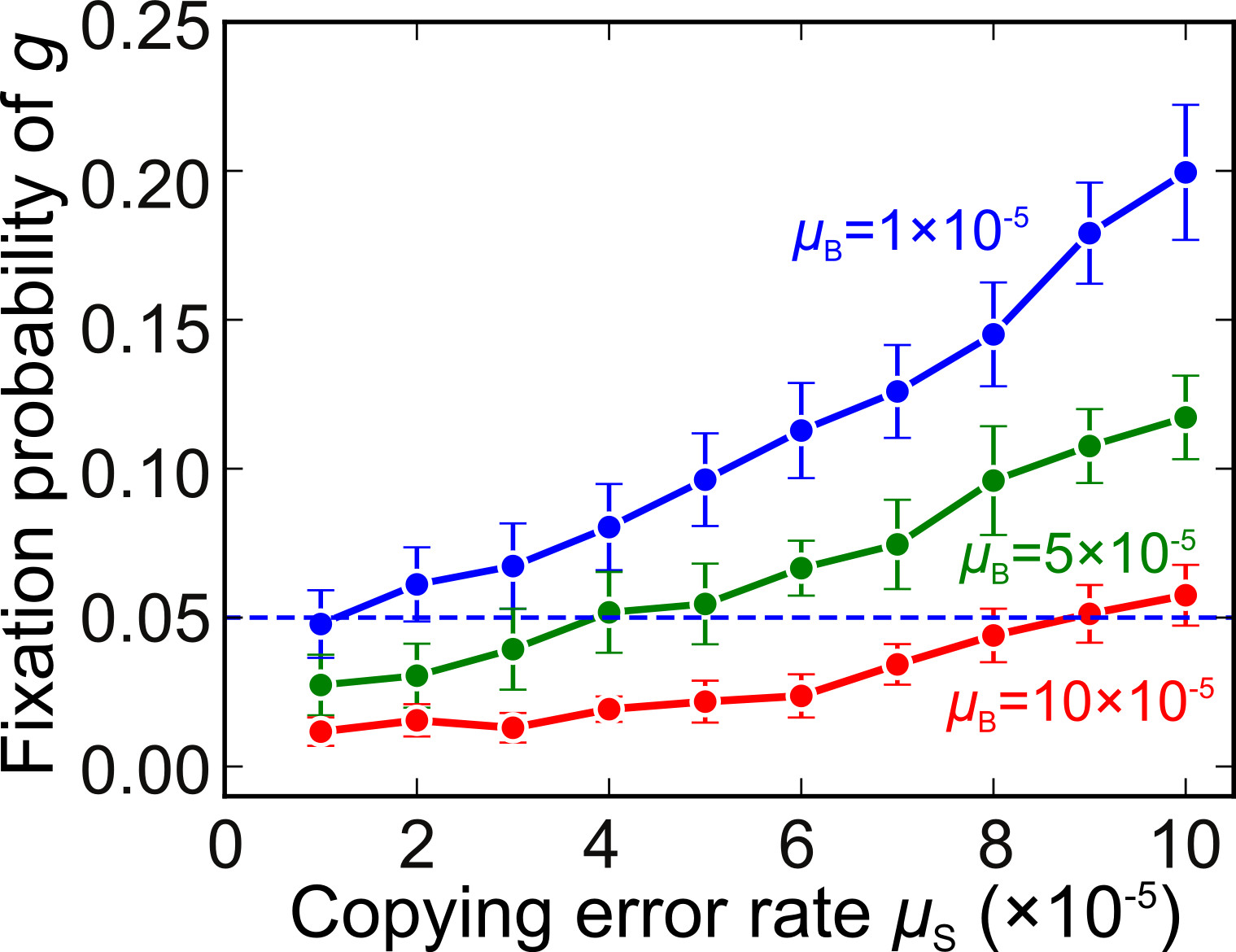

Supplement: S3 Fig — Fixation probability of an allele encoding early germline sequestration (at generation NG = 10) in an organism with an extended life cycle that produces gametes by somatic gametogenesis at generation NG = 60. The number of somatic cell divisions is significantly higher than in the simulations used in the main text where we considered invasion of an early germline at generation NG = 3 for an organism with a life cycle of NG = 10 generations. With the extended life cycle there is a much higher opportunity for the build-up of segregational variation and as a result early germline sequestration is favoured with mitochondrial mutation rates that are ~2 orders of magnitude lower. Once again, early germline sequestration is favoured by low background mutation rates (μB) and high replication error rates (μS). To reduce computational complexity, the adult fitness of an organism has been approximated according to the probability distribution function p(60) as defined in the Methods, while the number of mutant mitochondria in gametes is drawn from probability distribution functions p(60) and p(10). The number of mitochondria per cell is M = 50, selection strength s = 1, with a single tissue. The dashed line indicates the fixation probability of a neutral mutant. Underlying data can be found at: https://github.com/ArunasRadzvilavicius/GermlineEvolution/tree/master/FigureData. (PNG) [file pbio.2000410.s003.png]

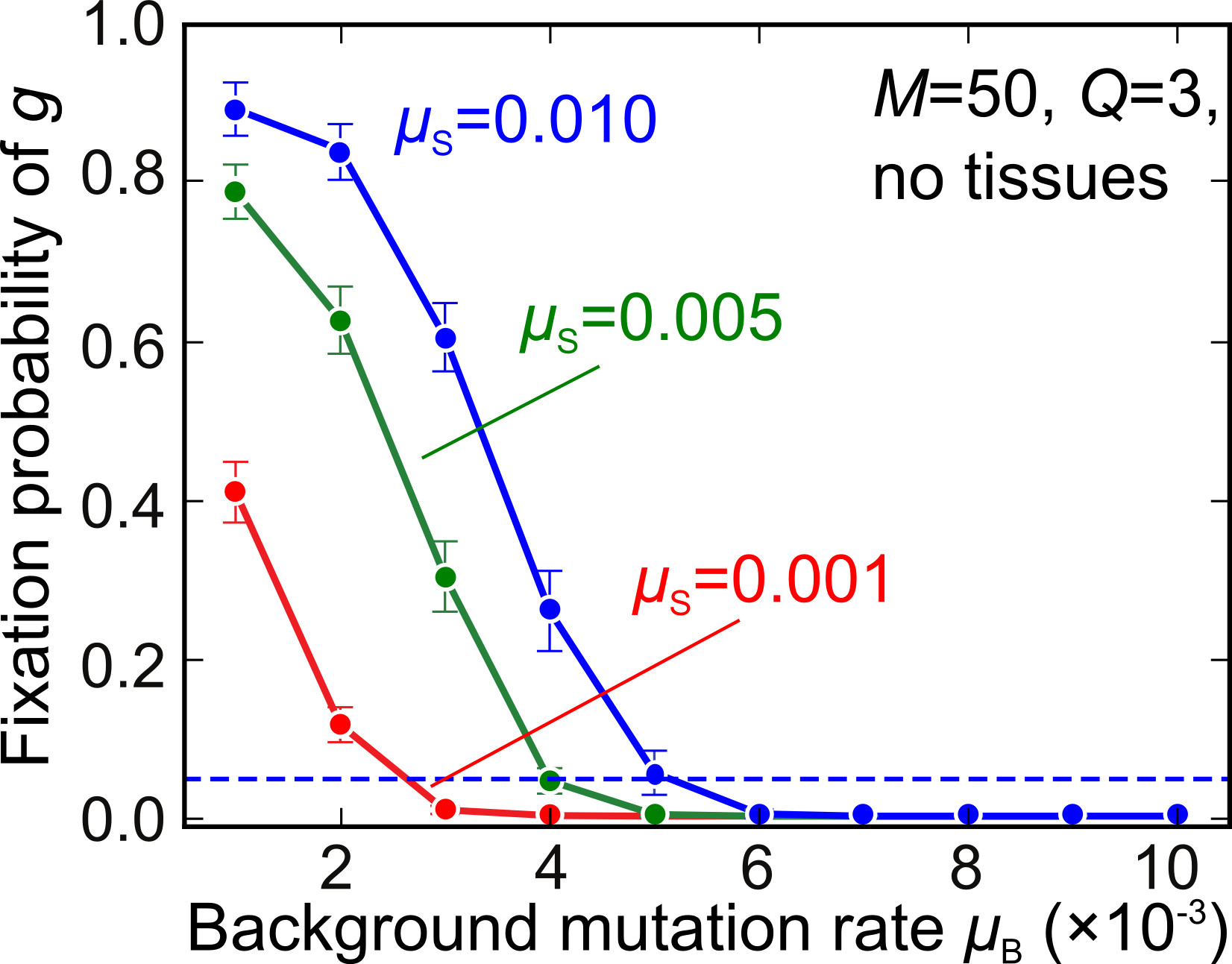

Supplement: S4 Fig — The evolution of early germline sequestration (at generation NG = 3) and oogamy (Q = 3) is favoured by low background mutation rates (μB). The allele A specifying early germline sequestration is more likely to be fixed at higher values of mutation due to copying errors (μS). Germline fixation also depends on the background mutation rate (μB). Germline sequestration does not fix if μB > 6 x 10^−3^ but fixes readily at lower μB, even when μS is low. Adaptations that favour low μB are characteristic of bilaterians germlines, including sequestration of oocytes in an internal ovary, repression of transcription and translation, and suppression of mitochondrial respiration [17,21]. The dashed line indicates the fixation probability of a neutral mutant. Underlying data can be found at: https://github.com/ArunasRadzvilavicius/GermlineEvolution/tree/master/FigureData. (PNG) [file pbio.2000410.s004.png]
